# Validation of an instrument for assessing leprosy care in children and adolescents

**DOI:** 10.1590/0034-7167-2023-0344

**Published:** 2024-07-19

**Authors:** Gutembergue Santos de Sousa, Isael Marcos Silva Mendonça, Lia Hanna Martins Morita, Denise da Costa Boamorte Cortela, Pãmela Rodrigues de Souza Silva, Silvana Margarida Benevides Ferreira

**Affiliations:** I Universidade Federal do Mato Grosso. Cuiabá, Mato Grosso, Brazil; II Faculdade para o Desenvolvimento Sustentável da Amazônia. Parauapebas, Pará, Brazil; III Universidade do Estado do Mato Grosso. Cáceres, Mato Grosso, Brazil

**Keywords:** Validation Studies, Leprosy, Child, Adolescent, Primary Health Care, Estudio de Validación, Lepra, Niño, Adolescente, Atención Primaria de Salud

## Abstract

**Objectives:**

to validate the content of an instrument for assessing leprosy care in individuals under 15 years old in the context of Primary Health Care.

**Methods:**

methodological study of content validation, based on the evaluation of essential and derived attributes in primary care, in the professional version. For data analysis, the Content Validation Index (CVI ≥ 0.8) and Cronbach’s Alpha were calculated.

**Results:**

a higher percentage of judges among nurses (61.5%) was observed; with a doctorate (46.2%), and engaged in teaching and research (77%). The overall Content Validation Index of the instrument was 0.98. In the analysis of Cronbach’s Alpha of the instrument, the assigned value was 0.717.

**Conclusions:**

the instrument represents an advancement in the measurement of health evaluation policies and can significantly contribute to improving the quality of care provided to children and adolescents with leprosy.

## RESUMEN

**Objetivos:**

validar el contenido de un instrumento para evaluar la atención a la lepra en menores de 15 años en el contexto de la Atención Primaria de la Salud.

**Métodos:**

estudio metodológico de validación de contenido, según la evaluación de atributos esenciales y derivados en la atención primaria, en su versión profesional. Para el análisis de los datos, se calcularon el Índice de Validación de Contenido (IVC ≥ 0,8) y el Alfa de Cronbach.

**Resultados:**

se observó un mayor porcentaje de jueces entre enfermeros (61,5%); con doctorado (46,2%), y que trabajaban en enseñanza e investigación (77%). El Índice de Validación de Contenido general del instrumento fue de 0,98. En el análisis del Alfa de Cronbach del instrumento, se atribuyó un valor de 0,717.

**Conclusiones:**

el instrumento representa un avance en la medición de políticas evaluativas de salud y puede contribuir significativamente a la mejora de la calidad de la atención prestada a niños y adolescentes con lepra.

## INTRODUCTION

Leprosy, as an infectious, chronic, and neglected tropical disease, is present in 127 countries worldwide, and despite all efforts for its elimination, remains a significant public health problem, particularly in Brazil, which ranks as the second country with the highest number of cases globally^(1)^. The incidence of leprosy in individuals under 15 years old represents an active factor in community disease transmission and should be continuously monitored. In 2021, 9,052 new cases of the disease were detected in this population globally, accounting for 4.49% of the total of 140,594 cases, with a detection rate of 17.83 per million inhabitants^(1)^. However, with the advent of the Covid-19 pandemic, a reduction in the number of cases by 37.1% was observed compared to the years 2019 and 2020, which may indicate possible underreporting of cases globally. Given this magnitude, evaluating the care provided to these individuals, especially children, can guide the implementation of better strategies for tackling the disease, directly impacting quality of life and social relations^(1-3)^.

Studies on leprosy in individuals under 15 years old in Brazil demonstrate the need for a more effective direction of public health policies, particularly at the primary care levels, contributing to the understanding of the endemic behavior of the disease^(4)^. In this aspect, health quality, as a necessary construct for quality of life, encompasses various perspectives, including clinical and population-based. From this perspective, the impact of actions on individual health and existing disparities in the health of population subgroups are evaluated.

Thus, health quality and quality in health care need to be aligned to create mechanisms that ensure universal access within the principles of comprehensiveness in care provision^(4-5)^. Primary Health Care (PHC), in this scenario, is characterized by a set of actions aimed at developing comprehensive, quality care that aims to enhance people’s autonomy and their health status individually and collectively, through the integration of health care services based on the essential and derived attributes of PHC^(5)^.

This orientation follows structuring axes recognized in Brazilian^(5)^ and international literature^(6)^, with the essential attributes including: Access/Attention, related to the first contact the user has with the health service for each new or recurring problem, considered the gateway to the health service; Longitudinality, referring to care offered regularly, requiring continuous attention over time; Comprehensiveness, which encompasses dimensions structured in actions of health promotion, prevention, and protection for the individual/family, as well as care at various levels of complexity of medical assistance; and Coordination, which can be seen as the articulation between various services and health actions in an integrated manner^(6)^.

According to the Primary Care Assessment Tool (PCATool), derived attributes presuppose a qualification of the care provided, based on aspects related to family and community orientation, as well as cultural competence in the process of health care^(5-6)^.

The Brazilian healthcare system faces the challenge of maintaining and ensuring the quality of health services for individuals affected by diseases, regardless of age, sex, place of residence, or origin. This implies ensuring equal opportunities for diagnosis and appropriate and timely treatment at all levels of care^(7-8)^.

In this context, health service evaluation is crucial as a decision-making process based on scientific evidence, capable of guiding and/or modifying service provision to ensure an adequate response to the population’s health demands. This allows for the reformulation of practices through managerial competence and the incorporation of information production to define new intervention strategies^(9)^.

Validating a measurement instrument means ensuring that it can truly measure what it proposes through the evaluation of its psychometric properties, thus ensuring the reliability of its use^(10-11)^. The lack of validated instruments to assess the care of children and adolescents with leprosy becomes an obstacle in healthcare services and disease monitoring in this age group. From this perspective, the present study addressed the validation of the Instrument for Evaluating the Quality of Care for Children and Adolescents with Leprosy in the PHC Context as a managerial tool in the process of evaluating leprosy services.

## OBJECTIVES

To validate the content of an instrument for evaluating leprosy care in children and adolescents within the context of Primary Health Care (PHC).

## METHODS

### Ethical Considerations

This study received approval from the Research Ethics Committee of the University of Cuiabá, conducted in accordance with Resolution number 466/2012 of the National Health Council. All participants provided their informed consent.

### Study Design, Period, and Location

This is a methodological study, grounded in PHC attributes^(5-6,12)^, guidelines for effective leprosy care^(13)^, and the Brazilian National Primary Care Policy (PNAB)^(14)^. The research aimed to develop and validate the content of the questionnaire titled “Instrument for Evaluating the Quality of Care for Children and Adolescents with Leprosy in the PHC Context”, targeting health professionals (physicians and nurses), considering the evaluation criteria of judges in the validation process.

The instrument comprises questions including the identification of the interviewee and aspects of leprosy care in individuals under 15 years old. It encompasses 8 domains totaling 95 questions (items), covering: entry point (5 items), access (9 items), continuous care (9 items), comprehensiveness of available and provided services (36 items), care coordination (17 items), family orientation (7 items), community orientation (7 items), and professional orientation (5 items). Each domain reflects an essential or derived attribute of PHC.

For each item of the instrument, a dichotomous scale was used (1. Yes; 2. No) or a Likert-type scale with the options: 1. Definitely not; 2. Probably not; 3. Probably yes; 4. Definitely yes; 9. I don’t know/I don’t remember.

Data collection for content validation occurred between September and November 2020. Judges were selected based on their specialization and willingness to participate in the study, representing various regions of the country.

### Population or Sample; Inclusion and Exclusion Criteria

The population was defined by selecting a panel of experts, chosen based on one of the following criteria: having a minimum of two years’ experience in teaching or research in leprosy or PHC; or having a minimum of two years’ experience working in PHC with leprosy cases.

Forty-five professionals were selected to participate in the study based on their *Lattes* resumes (https://lattes.cnpq.br/) and professional experience, following convenience sampling criteria. Of these, 13 professionals responded to the invitation and agreed to participate in the proposed content validation phase of this study. Experts who did not respond to the second call were excluded from the study.

### Study Protocol

The initial version of the instrument comprised 97 items distributed across domains (essential and qualifying attributes of PHC)^(5-6,12)^: 1. Entry point; 2. Access; 3. Continuous care; 4. Comprehensiveness of available and provided services; 5. Coordination; 6. Family orientation; 7. Community orientation; 8. Professional orientation.

To assess each attribute, specialists responded to two questions:

1. Is the content of this question essential for evaluating the organizational/assistance aspects of PHC in leprosy care for children and adolescents? If “NO,” please provide justification.
2. Is the content of this item appropriate for the respective PHC attribute it belongs to?

The first question was answered dichotomously (1. Yes; 2. No), while the second question used a Likert scale (1. Not suitable; 2. Very slightly suitable; 3. Slightly suitable; 4. Suitable; 5. Very suitable).

For content validation, the Delphi Technique was employed, following the primary methodological principles for health research^(15-17)^. Two rounds of instrument evaluation were conducted until consensus was reached among specialists, as defined by the technique.

In the initial panel, 15 judges assessed the instrument. Due to the lack of consensus and various suggestions for inclusion, exclusion, and item modification, a second panel was convened with the same specialists, taking into account the suggestions presented. In this subsequent panel, only 13 judges provided evaluations, reaching consensus with the implemented changes, thereby concluding the specialist panel.

Following evaluation, 6 items deemed essential for assessing leprosy care in individuals under 15 years old were included. Eight items considered redundant or unnecessary were excluded, and 7 items underwent wording adjustments to enhance understanding and comprehension by the judges, resulting in a total of 95 items with validated content.

### Results Analysis and Statistics

Robust statistical measures were employed to evaluate and validate the collected data. Initially, the Content Validation Index (CVI) was calculated based on responses to question 01 of the research instrument. Content validity was deemed satisfactory when the CVI was equal to or greater than 0.80, indicating acceptable agreement among evaluators, as per the adopted methodological reference^(17-18)^.

Furthermore, to assess the internal consistency of responses to question 02, Cronbach’s Alpha coefficient was calculated using Statistical Package for the Social Science (SPSS) version 20. This analysis enabled verification of response reliability and internal measure coherence, thereby bolstering the study’s results.

## RESULTS

The sample characterization of the participating judges in this study reveals a diversified representation, predominantly composed of nurses, totaling 61.5% (n=8), followed by physicians, who represented 23.1% (n=3) of the group. Regarding academic qualifications, a predominance of doctorate degrees is observed, comprising 46.2% (n=6) of the judges, followed by master’s degree professionals, who constituted 30.8% (n=4) of the total. Concerning the area of expertise, the majority of judges, accounting for 77% of the group, are involved in teaching and research (n=10), demonstrating a significant academic contribution to the validation of the essential and derived attributes of PHC in the care of individuals under 15 years old with leprosy, as presented in Table 1.

**Table 1:** Characterization of the judges participating in the content validation of the essential and derived attributes of Primary Health Care in the care of individuals under 15 years old with leprosy (N=13), Brazil, 2020

	n	%
Professional Category		
Nurse	8	61.5
Physician	3	23.1
Physiotherapist	2	15.4
Total	13	100
Academic Degree		
Specialist	3	23.1
Master	4	30.8
Doctor	6	46.2
Total	13	100
Area of Expertise		
Teaching	5	38.5
Research	5	38.5
Assistance	3	23.1
Total	13	100

It can be observed in Table 2 that all attributes obtained Content Validity Index (CVI) values above the established cutoff point (CVI ≥ 0.80), with the lowest CVI value, 0.94, recorded for the Access attribute. Additionally, it is noted that the Entry Point and Community Orientation attributes achieved a CVI of 1. The total CVI value for the instrument was 0.98, with a Cronbach’s Alpha of 0.717.

**Table 2:** Distribution of items of the essential and derived attributes in the content validation, before and after the judges’ evaluation, in the care of individuals under 15 years old with leprosy in Primary Health Care, Brazil, 2022

Atributos	Number of Items	Item a	Item b	Item c	CVI	Cronbach's Alpha
Entry Point	5	-	-	-	1.00	
Access	11	3	1	4	0.94	
Continuous Care	9	-	1	3	0.98	
Comprehensiveness of available services	9	-	1	-	0.97	
Comprehensiveness of provided services	27	3	1		0.97	
Care Coordination	16	1	2	-	0.98	
Family Orientation	8	1	-	-	0.97	
Community Orientation	7	-	-	-	1.00	
Professional Orientation	5	-	-	-	0.98	
Total	97	8	6	7	0.98	0,717

*Item A - Items Included; Item B - Items Excluded; Item C - Items Modified; CVI - Content Validity Index*

The Chart 1 details the items that were excluded and included as per the judges’ evaluation. Eight items related to the attributes Access, Continuous Care, Comprehensive Services Available and Provided, Care Coordination, and Family Orientation were excluded. The excluded items already had their contents represented in other aspects of the instrument; their absence did not compromise the integrity of the attribute to which they belonged, or their content lacked legal basis or was not clearly defined in health policies for leprosy.

**Chart 1:** Description of excluded and included items in the evaluation of essential and derived attributes, in the content validation process by judges, Brazil, 2022

Excluded items	
**Attribute**	**Item**
Access	Does the health facility open at least once on a Saturday or Sunday per month?
	During the operating hours of the PHC health facility, is there a telephone number available for users to request information?
	Can users schedule an appointment at the PHC health facility for supervised dosing?
Continuous Care	Do you respond to questions in a way that parents or caregivers of the child/adolescent can understand?
Comprehensiveness of services provided	Chemoprophylaxis with Rifampicin for contacts of children/adolescents with leprosy.
	Utilization of rapid serological tests for screening for Mycobacterium leprae infection in communities where cases of leprosy occur in children/adolescents.
Care Coordination	When the child/adolescent presents any health problems related to leprosy (examples: neuritis, medication reactions, leprosy reactions, psychological or social problems), can they receive care from a specialist in medium or high complexity?
Family Orientation	Do you discuss self-care techniques for preventing disabilities with the parents/caregivers of the child/adolescent?
**Included items**	
**Attribute**	**Item**
Access	Does the child/adolescent with leprosy miss school to attend the health facility?
Continuing care	Do you ask parents/caregivers/adolescents if people exhibit prejudice because of leprosy?
Comprehensiveness of available services	Prenatal care for pregnant adolescents.
Comprehensiveness of provided services	Dental follow-up for children/adolescents with leprosy.
Care coordination	Does the PHC health facility develop an Annual Work Plan specific to leprosy or utilize another similar management tool?

On the other hand, five items related to the attributes Access, Continuous Care, Comprehensive Services Available and Provided, and Care Coordination were included. Most of the added items were broken down from other items, allowing the information to be evaluated separately, minimizing the risk of bias. Some items were included because they were considered essential in evaluating leprosy care actions.

Chart 2 presents a detailed description of the items that underwent changes in their wording, according to the respective attributes that best fit the evaluative context. It is observed that modifications to the instrument’s items were made due to the need to separate some items to facilitate understanding and comprehension, as well as changes to terms and expressions more appropriate to the evaluative context. Only the attributes Access, Continuous Care, and Family Orientation underwent content changes in their items.

**Chart 2:** Description of essential and derived attributes in the content validation by judges, according to original and modified items, Brazil, 2022

Attribute	Original Item	Modified Item
Access	The APS health facility remains open after 6:00 PM at least one day during the week.	Does the APS health facility remain open during lunchtime or after 6:00 PM at least one day during the week?
	Do parents/caregivers/adolescents face transportation difficulties or need to use some form of motorized transport to reach the APS health facility?	Do parents/caregivers/adolescents need to use any form of motorized transportation, due to distance, to reach the APS health facility?
	Do parents/caregivers of the person with leprosy miss work shifts or appointments, causing the child/adolescent to miss school to be seen at the health facility?	Do parents/caregivers miss work or appointments so that the child/adolescent with leprosy can be seen at the health facility?
	Does the health facility have means to communicate with the user/patient (e.g., telephone, SMS text messages, messaging apps like WhatsApp, email, etc.) about treatment, appointment confirmations, etc.?	Does the health facility have means to communicate with the user/patient (e.g., telephone, SMS text messages, messaging apps like WhatsApp, email, etc.) about treatment, appointment confirmations, etc.?
Continuous care	Are you familiar with the entire health history of the child/adolescent with leprosy?	Does the health facility have means to communicate with the user/patient (e.g., telephone, SMS text messages, messaging apps like WhatsApp, email, etc.) about treatment, appointment confirmations, etc.?
	Do you ask parents/caregivers of the child/adolescent how leprosy impacts daily life, if there is prejudice from others, or if the disease affects school and leisure activities?	Do you inquire with parents/caregivers/adolescents about how leprosy affects daily life and interferes with school and leisure activities?
Family orientation	Do you discuss self-care techniques for preventing disabilities with parents/caregivers of the child/adolescent?	Do you provide guidance to parents/caregivers of the child/adolescent about the signs and symptoms of leprosy reactions, as well as instructing them on self-care techniques for preventing disabilities?

## DISCUSSION

The content validation process is crucial in developing measures and instruments in the healthcare field, enabling safer evaluation and practice, free from measurement errors. This process encompasses theoretical concepts and practical aspects linked to the judgment and perception of a group of experts with prior knowledge in the subject to be validated, with the ultimate goal of obtaining an instrument capable of precisely measuring what is proposed^(19)^.

The validation of the Instrument for Evaluating the Quality of Care for Children and Adolescents with Leprosy in the Context of PHC, proposed in this research, establishes an important milestone for the evaluation of essential and derived attributes in PHC. The instrument was evaluated by judges with expertise in the subject, predominantly nurses, with a doctorate and experience in teaching and research.

Regarding the evaluated items, according to the essential and derived attributes, the total Content Validation Index (CVI) was 0.98, and the Entry Point and Community Orientation attributes obtained the highest scores, ensuring satisfactory values that guarantee validity parameters regarding the content used for the development of the items of each attribute of the instrument.

Content validation analyses reflect an essential process in the development of service evaluation instruments, especially in more vulnerable populations. The increase in leprosy cases in this age group indicates the endemic persistence of the condition in the community. This procedure is vital to avoid inaccurate or biased results and measures that may lead to erroneous or poorly measured conclusions.

Regarding the sample of judges for instrument evaluation in health services, it is advocated that this sample be multiprofessional because professionals from different areas can contribute diverse perspectives, ideas, and experiences, increasing rigor, criteria, and objectivity in evaluating the phenomenon and validated content^(20)^. In this study, the judges were mainly nurses and physicians, who are most often the professionals providing leprosy care in PHC and therefore have greater capacity to evaluate the essential and derived attributes in serving the studied population.

Regarding the number of judges for the Delphi technique in content validation, various institutions and authors agree that the appropriate minimum number should be at least seven specialists, as this reduces the probability of errors in measurement. It is also mentioned that the maximum number of judges should not exceed thirty participants, as the benefits observed with a larger number are not significant. Thus, the number of judges used in this study was considered satisfactory, falling within the minimum and maximum recommended by the literature^(21-22)^.

As for the fact that the sample of judges consisted of specialists, masters, and doctoral professionals working as teachers, researchers, or healthcare workers, it is relevant to highlight that the choice was based on professionals with experience in the topic of interest, considerably reducing possible inconsistencies in the results^(23-25)^.

The validation of the healthcare professionals’ version of the instrument for assessing the performance of primary healthcare (PHC) in leprosy control actions in the adult population (PCAT - leprosy) supports the findings of the present study, indicating satisfactory values in terms of validity parameters, internal consistency, and reliability, ensuring its utility in health service evaluation processes^(12)^.

Validating instruments that utilize PHC attributes for service evaluation is crucial, both from a general perspective and in specific diseases. There is a need to measure the degree of service orientation within a system that prioritizes PHC in the care model, focusing on evaluative parameters that identify aspects requiring greater investment or the reorientation of actions in the healthcare system^(26)^.

The Entry Point attribute, in this study, demonstrated a satisfactory score in content validation, being considered essential as a component of first-contact access, crucial for the interface between users and healthcare services, covering spontaneous demands and appointment scheduling^(27-29)^.

Investing in tools and actions that facilitate users’ access to healthcare services, especially in the child population, is vital. In this perspective, access and accessibility to services, such as leprosy prevention, active case finding, chemoprophylaxis, early diagnosis, screening using innovative molecular biology techniques, and timely treatment, are fundamental^(30-32)^.

Moreover, issues related to waiting times for care, geographic distance from healthcare facilities, operating hours, and shifts, which could be aligned with the operation of daycare centers and educational institutions, are essential to enhance more qualified assistance. In this perspective, Access is seen as the main essential attribute of PHC, acting as the bridge between the system and the user, ensuring equity and quality in service provision^(33)^.

Regarding Continuity of Care or Continuous Care, the literature highlights the great potential of this attribute within the PHC context, as it enables a deep understanding of the user in their social context, life history, habits, and customs. This allows for planning and establishing individual or collective care plans, providing appropriate and conclusive interventions. It is observed that the Family Health Strategy (FHS) presents better values in the assessment of Continuous Care compared to traditional primary healthcare units, being considered a care model that promotes longitudinal care, which can positively impact the quality of healthcare provided^(34-35)^.

The comprehensiveness of Available and Provided Services, as a PHC attribute, aims to value the quality of care systematization, especially in the leprosy care protocol for children and adolescents, considering also the biopsychosocial aspects associated with this clientele^(5,36-39)^.

On the other hand, the Care Coordination attribute aims to establish connections in a service network within the healthcare system, achieving Comprehensive Care and promoting Health Care Continuity. This attribute strengthens aspects that favor service quality and care provision, aiming to reduce access barriers to different levels of care and integrate as many services as possible within the same territory, even in diverse contexts. Care Coordination instrumentalizes mechanisms that correlate the organization of healthcare networks in a regionalization process, seeking to improve processes through queue reduction, prioritization of needs, and the development of protocols and flowcharts aimed at standardizing service quality^(40-41)^.

Additionally, studies aimed at validating instruments for assessing care coordination and organization in care networks by PHC demonstrate that the majority of proposed items achieved a sufficient degree of stability, with high consensus among the specialists involved in the content validation process, highlighting the importance of items that evaluate the continuity of health actions provided by different professionals at different points in the care network^(42-43)^.

Finally, Family Orientation, as an attribute of PHC, should be based on a family-centered care model, allowing for an expanded analysis of issues related to the health-disease process through a socio-spatial hermeneutic, focused on service reorganization and especially directed toward the FHS as the coordinator of family care, respecting the principles of universality, equality, and equity of the healthcare system. In the context of child and adolescent health, this attribute is even more relevant because the care for this clientele is influenced by the family’s perception of promotion, prevention actions, and the disease process^(44-45)^.

The community’s approach to health services and decisions is a major challenge to be overcome in the context of PHC. It is imperative to find mechanisms for PHC units to be community-oriented, establishing a connection between the service, healthcare professionals, and users present in the geographical space or social and political context that defines the territory^(36,46)^.

In this context, professional orientation aims to understand professionals’ perception of their own qualifications in addressing health problems, being a central phenomenon for evaluation. This attribute encompasses crucial issues related to professional training and continuing education. Thus, the importance of investing in the training and capacity building of physicians, nurses, and other healthcare professionals who serve children and adolescents is emphasized. It is vital to establish partnerships with educational institutions, promote professional valorization, improve the physical structure of services, and invest in innovative technological tools for implementing care practices^(12,47)^.

In the validation of the PCATool Brazil version for adults, it is observed that the content validation process did not adopt the Delphi technique, preventing comparisons between the two models^(48)^.

The instrument in question was employed in a study in a municipality in the state of Pará, proving to be an adequate tool to fulfill its measurement purpose^(49)^.

### Study limitations

This study has limitations. Firstly, the sample of judges, although qualified and diverse, is limited in number and may not represent all perspectives and experiences of healthcare professionals working in PHC in different regions and contexts. Additionally, the content validation process, while rigorous, may have been influenced by the judges’ subjectivity in interpreting the instrument items.

### Contributions to Nursing and Public Health

Despite the limitations, content validity is fundamental in the process of developing and adapting measurement instruments. The inclusion of validated questions about health issues or policies in the official assessment instruments of the Ministry of Health represents a significant advancement in the pursuit of adequate measurement in the country’s health evaluation policy. Considering that health assessment is a necessary measure in the process of improving the quality of care, the instrument validated for the proposed content in this study can contribute satisfactorily to the evaluation of actions in leprosy care in the context of PHC, especially in the care of children and adolescents with leprosy.

## CONCLUSIONS

This study aimed to validate the content of the instrument for assessing the quality of care for children and adolescents with leprosy in the context of Primary Health Care. The results obtained demonstrate that the proposed instrument achieved a high level of content validity, as indicated by the Content Validation Index and satisfactory reliability values, measured by Cronbach’s Alpha.
