# Supplementary material for: Validation of an instrument for assessing leprosy care in children and adolescents
Source: Rev Bras Enferm. 2024 Jul 19;77(2):e20230344. doi: 10.1590/0034-7167-2023-0344 (PMC11259440; doi:10.1590/0034-7167-2023-0344)
Supplement: Translation

# Translation: Validação de um instrumento para avaliação da assistência em hanseníase em crianças e adolescentes

**DOI:** 10.1590/0034-7167-2023-0344pt

**Authors:** Gutembergue Santos de Sousa, Isael Marcos Silva Mendonça, Lia Hanna Martins Morita, Denise da Costa Boamorte Cortela, Pãmela Rodrigues de Souza Silva, Silvana Margarida Benevides Ferreira

**Affiliations:** I Universidade Federal do Mato Grosso. Cuiabá, Mato Grosso, Brasil; II Faculdade para o Desenvolvimento Sustentável da Amazônia. Parauapebas, Pará, Brasil; III Universidade do Estado do Mato Grosso. Cáceres, Mato Grosso, Brasil

**Keywords:** Estudos de Validação, Hanseníase, Criança, Adolescente, Atenção Primária à, Saúde

## Abstract

**Objetivos:**

validar o conteúdo de um instrumento para a avaliação da assistência à hanseníase em menores de 15 anos no contexto da Atenção Primária à Saúde.

**Métodos:**

estudo metodológico de validação de conteúdo, segundo a avaliação de atributos essenciais e derivados na atenção primária, na versão profissional. Para a análise dos dados, foram calculados o Índice de Validação de Conteúdo (IVC ≥ 0,8) e o *Alfa* de Cronbach.

**Resultados:**

observou-se um maior percentual de juízes entre enfermeiros (61,5%); com doutorado (46,2%), e que atuavam no ensino e na pesquisa (77%). O Índice de Validação de Conteúdo geral do instrumento foi de 0,98. Na análise do Alfa de Cronbach do instrumento, o valor atribuído foi de 0,717.

**Conclusões:**

o instrumento representa um avanço na mensuração de políticas avaliativas de saúde e pode contribuir significativamente para a melhoria da qualidade da atenção prestada a crianças e adolescentes com hanseníase.

## INTRODUÇÃO

A hanseníase, enquanto doença infecciosa, crônica e tropical negligenciada, está presente em 127 países pelo mundo e, apesar de todos os esforços para sua eliminação, continua sendo um problema de saúde pública, principalmente no Brasil, que é o segundo país com o maior número de casos no mundo^(1)^. A incidência da hanseníase em menores de 15 anos representa um fator ativo de transmissão comunitária da doença e deve ser monitorada continuamente. Em 2021, foram detectados, nessa população, 9.052 novos casos da doença no mundo, correspondendo a 4,49% do total de 140.594 casos, com uma taxa de detecção de 17,83 por milhão de habitantes^(1)^. Entretanto, com o advento da pandemia de Covid-19, observou-se uma redução no número de casos de 37,1% em comparação aos anos de 2019 e 2020, o que pode representar uma possível subnotificação dos casos registrados globalmente. Diante dessa magnitude, a avaliação da assistência prestada a essas pessoas, principalmente às crianças, pode direcionar a implementação de melhores estratégias para o enfrentamento da doença, impactando diretamente na qualidade de vida e nas relações sociais^(1-3)^.

Estudos sobre a hanseníase em menores de 15 anos no Brasil demonstram a necessidade de um direcionamento mais efetivo das políticas públicas de saúde, principalmente nos níveis primários de atenção, contribuindo para a compreensão do comportamento endêmico da doença^(4)^. Nesse aspecto, a qualidade em saúde, enquanto um construto necessário para a qualidade de vida, abrange diversas perspectivas, entre elas a clínica e a populacional. Sob essa perspectiva, avalia-se o impacto das ações sobre a saúde individual e sobre as disparidades existentes na saúde de subgrupos populacionais^(5)^.

Assim, a qualidade em saúde e a qualidade na atenção à saúde precisam estar alinhadas de modo a criar mecanismos que garantam o acesso universal dentro dos preceitos da integralidade no atendimento^(4-5)^. A Atenção Primária à Saúde (APS), nesse cenário, caracteriza-se por um conjunto de ações com o objetivo de desenvolver uma atenção integral, de qualidade, que direcione para elevar a autonomia das pessoas e sua situação de saúde de maneira individual e coletiva, por meio da inserção nos serviços de uma atenção em saúde pautada nos atributos essenciais e derivados da APS^(5)^.

Essa orientação segue eixos estruturantes reconhecidos na literatura brasileira^(5)^ e internacional^(6)^, sendo os atributos essenciais o acesso de primeiro contato, longitudinalidade, integralidade, coordenação do cuidado e, os derivados: orientação familiar, comunitária e competência cultural^(5)^.

Os atributos essenciais, conforme definidos pelo *Primary Care Assessment Tool* (PCATool), incluem: o Acesso/Atenção, relacionado ao primeiro contato que o usuário tem com o serviço de saúde para cada problema novo ou recorrente, considerado a porta de entrada para o serviço de saúde; a Longitudinalidade, que se refere aos cuidados ofertados de forma regular, exigindo uma atenção contínua ao longo do tempo; a Integralidade, que abrange as dimensões estruturadas nas ações de promoção, prevenção e proteção à saúde do indivíduo/família, além da atenção nos diversos níveis de complexidade da assistência médica; e a Coordenação, que pode ser vista como a articulação entre os diversos serviços e ações de saúde de maneira integrada^(6)^.

De acordo com o PCATool, os atributos derivados pressupõem uma qualificação da atenção prestada, a partir de aspectos relacionados à orientação familiar, comunitária e à competência cultural no processo de assistência em saúde^(5-6)^.

O sistema de saúde brasileiro enfrenta o desafio de manter e assegurar a qualidade dos serviços de saúde para pessoas afetadas por doenças, independentemente de idade, sexo, local de residência ou procedência. Isso implica garantir igualdade de oportunidades para diagnóstico e tratamento adequado e oportuno em todos os níveis de atenção^(7-8)^.

Neste contexto, a avaliação de serviços de saúde é crucial como um processo de tomada de decisão baseada em evidências científicas, capazes de direcionar e/ou modificar a prestação de serviços, assegurando uma resposta adequada às demandas de saúde da população. Isso permite a reformulação de práticas através da competência gerencial e da incorporação da produção de informação para definir novas estratégias de intervenção^(9)^.

Validar um instrumento de medida significa assegurar que este realmente possa mensurar o que se propõe, através da avaliação de suas propriedades psicométricas, garantindo assim a confiabilidade de seu uso^(10-11)^. A falta de instrumentos validados para avaliar a assistência à criança e ao adolescente com hanseníase torna-se um obstáculo no serviço de saúde e no monitoramento da doença nesta faixa etária. Sob esta ótica, o presente estudo abordou a validação do Instrumento para Avaliação da Qualidade da Atenção à Criança e ao Adolescente com Hanseníase no Contexto da APS, como uma ferramenta gerencial no processo de avaliação de serviços em hanseníase.

## OBJETIVOS

Validar o conteúdo de um instrumento para avaliação da assistência em hanseníase em crianças e adolescentes no contexto da Atenção Primária à Saúde (APS).

## MÉTODOS

### Aspectos éticos

Este estudo foi aprovado pelo Comitê de Ética em Pesquisa da Universidade de Cuiabá, desenvolvido conforme a Resolução nº 466/2012 do Conselho Nacional de Saúde. Todos os participantes assinaram o Termo de Consentimento Livre e Esclarecido.

### Desenho, período e local do estudo

Trata-se de um estudo metodológico, embasado nos atributos da APS^(5-6,12)^, nas diretrizes para ações e estratégias eficazes da assistência em hanseníase^(13)^ e na Política Nacional da Atenção Básica (PNAB)^(14)^. A pesquisa teve como objetivo construir e realizar a validação de conteúdo do questionário denominado “Instrumento para Avaliação da Qualidade da Atenção à Criança e Adolescente com Hanseníase no Contexto da APS”, direcionado a profissionais de saúde (médicos e enfermeiros), levando em consideração os critérios de avaliação dos juízes no processo de validação.

O instrumento é composto por questões que incluem a identificação do entrevistado e aspectos da atenção à hanseníase em menores de 15 anos. Apresenta 8 domínios que totalizam 95 questões (itens), abrangendo: porta de entrada (5 itens), acesso (9 itens), atendimento continuado (9 itens), integralidade dos serviços disponíveis e prestados (36 itens), coordenação do cuidado (17 itens), orientação familiar (7 itens), orientação comunitária (7 itens) e orientação profissional (5 itens). Cada domínio reflete um atributo essencial ou derivado da APS.

Para cada item do instrumento, utilizou-se uma escala dicotômica (1. Sim; 2. Não) ou uma escala tipo Likert com as opções: 1. Com certeza, não; 2. Provavelmente, não; 3. Provavelmente, sim; 4. Com certeza, sim; 9. Não sei/não lembro.

A coleta de informações para a validação de conteúdo ocorreu entre setembro e novembro de 2020. Os juízes foram selecionados com base na sua especialização e aceitação para participar do estudo, representando diversas regiões do país.

### População ou amostra; critérios de inclusão e exclusão

A população foi definida pela seleção de um painel de especialistas, escolhidos com base em um dos seguintes critérios: ter uma experiência mínima de dois anos em docência ou pesquisa em hanseníase ou atenção primária à saúde; ou ter experiência mínima de dois anos de atuação na APS com atendimento a casos de hanseníase.

Foram selecionados 45 profissionais para participar do estudo com base em seus currículos *Lattes* (https://lattes.cnpq.br/) e experiência profissional, seguindo critérios de amostragem por conveniência. Desses, 13 profissionais responderam ao convite e aceitaram participar da fase de validação de conteúdo proposta neste estudo. Especialistas que não responderam até a segunda chamada foram excluídos do estudo.

### Protocolo do estudo

A primeira versão do instrumento continha 97 itens divididos entre os domínios (atributos essenciais e qualificadores da APS)^(5-6,13)^: 1. Porta de entrada; 2. Acesso; 3. Atendimento continuado; 4. Integralidade dos serviços disponíveis e prestados; 5. Coordenação; 6. Orientação familiar; 7. Orientação comunitária; 8. Orientação profissional.

Para a avaliação de cada um dos itens dos atributos analisados, os especialistas responderam duas questões:

1. O conteúdo desta questão é essencial para avaliar as características organizacionais/de assistência da APS na atenção à hanseníase em crianças e adolescentes? Se “NÃO”, justifique.
2. O conteúdo deste item está adequado ao respectivo atributo da APS a que está inserido?

A primeira questão era respondida de forma dicotômica (1. Sim; 2. Não), enquanto a segunda questão utilizava uma escala Likert (1. Não adequado; 2. Muito pouco adequado; 3. Pouco adequado; 4. Adequado; 5. Muito adequado).

Para a validação de conteúdo, foi adotada a Técnica Delphi, conforme os principais preceitos metodológicos para pesquisas na área da saúde^(15-17)^. Foram realizadas duas rodadas de avaliação do instrumento até que se alcançasse o consenso entre os especialistas, conforme definido pela técnica.

No primeiro painel, 15 juízes avaliaram o instrumento. Como não houve consenso e foram feitas várias sugestões de inclusão, exclusão e alteração de itens, realizou-se um segundo painel com os mesmos especialistas, considerando as sugestões apresentadas. Neste segundo painel, apenas 13 juízes apresentaram suas avaliações, alcançando consenso com as alterações realizadas, encerrando assim o painel de especialistas.

Após a avaliação, foram incluídos 6 itens considerados fundamentais para a avaliação da atenção à hanseníase em menores de 15 anos. Foram excluídos 8 itens considerados repetitivos ou desnecessários, e 7 itens tiveram sua redação alterada para melhorar a compreensão e o entendimento por parte dos juízes, resultando em um total de 95 itens com conteúdo validado.

### Análise dos resultados e estatística

Para a avaliação e validação dos dados coletados, foram utilizadas medidas estatísticas robustas. Inicialmente, o Índice de Validação de Conteúdo (IVC) foi calculado com base nas respostas à questão 01 do instrumento de pesquisa. A validade de conteúdo dos itens neste estudo foi considerada satisfatória quando o IVC era igual ou superior a 0,80, indicando uma concordância aceitável entre os avaliadores, conforme estabelecido pelo referencial metodológico adotado^(17-18)^.

Além disso, para avaliar a consistência interna das respostas à questão 02, foi calculado o coeficiente Alfa de Cronbach, utilizando o software estatístico *Statistical Package for the Social Science* (SPSS) versão 20. Esta análise permitiu verificar a confiabilidade das respostas e a coerência interna das medidas obtidas, agregando robustez aos resultados do estudo.

## RESULTADOS

A caracterização da amostra dos juízes participantes deste estudo revela uma representação diversificada, porém predominantemente composta por enfermeiros, totalizando 61,5% (n=8), seguidos por médicos, que representaram 23,1% (n=3) do grupo. Quanto à titulação, observa-se uma predominância de doutores, compreendendo 46,2% (n=6) dos juízes, seguidos por profissionais mestres, que constituíram 30,8% (n=4) do total. Em relação à área de atuação, a maioria dos juízes, correspondendo a 77% do grupo, está envolvida no ensino e na pesquisa (n=10), demonstrando uma significativa contribuição acadêmica para a validação dos atributos essenciais e derivados da Atenção Primária em Saúde (APS) na assistência a menores de 15 anos com hanseníase, conforme apresentado na Tabela 1.

**Tabela 1:** Caracterização dos juízes participantes na validação de conteúdo dos atributos essenciais e derivados da atenção primária em saúde na assistência a menores de 15 anos com hanseníase (N=13), Brasil, 2020

	n	%
Categoria Profissional		
Enfermeiro	8	61,5
Médico	3	23,1
Fisioterapeuta	2	15,4
Total	13	100
Titulação		
Especialista	3	23,1
Mestre	4	30,8
Doutor	6	46,2
Total	13	100
Área de Atuação		
Ensino	5	38,5
Pesquisa	5	38,5
Assistência	3	23,1
Total	13	100

Observa-se na Tabela 2 que todos os atributos obtiveram valores de Índice de Validade de Conteúdo (IVC) acima do ponto de corte estabelecido (IVC ≥ 0,80), sendo que o menor valor de IVC, de 0,94, foi registrado no atributo Acesso. Nota-se, também, que os atributos Porta de Entrada e Orientação Comunitária alcançaram IVC igual a 1. O valor total do IVC do instrumento foi de 0,98, com um Alfa de Cronbach de 0,717.

**Tabela 2:** Distribuição dos itens dos atributos essenciais e derivados na validação de conteúdo, antes e após a avaliação dos juízes, na assistência a menores de 15 anos com hanseníase na Atenção Primária à Saúde, Brasil, 2022

Atributos	Quantidade de itens	Item a	Item b	Item c	IVC	Alfa de cronbach
Porta de entrada	5	-	-	-	1,00	
Acesso	11	3	1	4	0,94	
Atendimento continuado	9	-	1	3	0,98	
Integralidade dos serv. disponíveis	9	-	1	-	0,97	
Integralidade dos serv. prestados	27	3	1		0,97	
Coordenação do cuidado	16	1	2	-	0,98	
Orientação familiar	8	1	-	-	0,97	
Orientação comunitária	7	-	-	-	1,00	
Orientação profissional	5	-	-	-	0,98	
Total	97	8	6	7	0,98	0,717

*Item A - Itens incluídos; Item B - Itens excluídos; Item C - Itens modificados; IVC - índice de validade de conteúdo.*

O Quadro 1 detalha os itens que foram excluídos e incluídos conforme a avaliação dos juízes. Foram excluídos oito itens relacionados aos atributos Acesso, Atendimento Continuado, Integralidade dos Serviços Disponíveis e Prestados, Coordenação do Cuidado e Orientação Familiar. Os itens excluídos já tinham seus conteúdos representados em outros aspectos do instrumento; sua ausência não comprometia a integralidade do atributo ao qual pertenciam, ou seu conteúdo não possuía embasamento legal ou não estava claramente definido nas políticas de saúde para a hanseníase.

**Quadro 1:** Descrição dos itens excluídos e incluídos na avaliação dos atributos essenciais e derivados, no processo de validação de conteúdo por juízes, Brasil, 2022

Itens Excluídos	
**Atributo**	**Item**
Acesso	A unidade de saúde abre pelo menos um sábado ou domingo por mês?
	Durante o horário de funcionamento da unidade de saúde da APS, há um número de telefone disponível para o usuário solicitar informações?
	O usuário pode agendar um horário na unidade de saúde da APS para realizar a dose supervisionada?
Atendimento Continuado	Você responde às perguntas de forma que os pais ou cuidadores da criança/adolescente compreendam?
Integralidade dos serviços prestados	Quimioprofilaxia com Rifampicina para os contatos de crianças/adolescentes com hanseníase.
	Utilização de testes rápidos sorológicos para rastreamento da infecção pelo *Mycobacterium leprae* na comunidade onde há ocorrência de casos de hanseníase em crianças/adolescentes.
Coordenação do cuidado	Quando a criança/adolescente apresenta algum problema de saúde relacionado à hanseníase (exemplos: neurite, reações medicamentosas, reações hansênicas, problemas psicológicos ou sociais), consegue atendimento com especialista em média ou alta complexidade?
Orientação familiar	Você dialoga com os pais/cuidadores da criança/adolescente sobre técnicas de autocuidado para a prevenção de incapacidades?
**Itens Incluídos**	
**Atributo**	**Item**
Acesso	A criança/adolescente com hanseníase falta às aulas para ser atendida na unidade de saúde?
Atendimento continuado	Você pergunta aos pais/cuidadores/adolescentes se as pessoas demonstram preconceito devido à hanseníase?
Integralidade dos serviços disponíveis	Atendimento pré-natal para as adolescentes grávidas.
Integralidade dos serviços prestados	Acompanhamento odontológico para crianças/adolescentes com hanseníase.
Coordenação do cuidado	A unidade de saúde da APS elabora o Plano Anual de Trabalho específico para a hanseníase ou utiliza outra ferramenta gerencial semelhante?

Por outro lado, foram incluídos cinco itens relacionados aos atributos Acesso, Atendimento Continuado, Integralidade dos Serviços Disponíveis e Prestados e Coordenação do Cuidado. A maioria dos itens adicionados foi desmembrada de outros itens, permitindo que as informações fossem avaliadas de forma separada, minimizando o risco de viés. Alguns itens foram incluídos por serem considerados essenciais na avaliação das ações de assistência à hanseníase.

O Quadro 2 apresenta o detalhamento dos itens que sofreram alterações em seu enunciado, de acordo com os respectivos atributos que melhor se adequam ao contexto avaliativo. Observa-se que as modificações nos itens do instrumento foram realizadas devido à necessidade de separar alguns itens para facilitar o entendimento e a compreensão, além de alterações de termos e expressões mais apropriados ao contexto avaliativo. Apenas os atributos Acesso, Atendimento Continuado e Orientação Familiar apresentaram mudanças de conteúdo em seus itens.

**Quadro 2:** Descrição dos atributos essenciais e derivados na validação de conteúdo por juízes, segundo itens originais e modificados, Brasil, 2022

Atributo	Item Original	Item Modificado
Acesso	A unidade de saúde da APS permanece aberta após as 18 horas pelo menos um dia durante a semana?	A unidade de saúde da APS permanece aberta durante o horário de almoço ou após as 18 horas pelo menos um dia durante a semana?
	Os pais/cuidadores/adolescentes enfrentam dificuldades de deslocamento ou necessitam usar algum tipo de transporte motorizado para chegar à unidade de saúde da APS?	Os pais/cuidadores/adolescentes precisam utilizar algum tipo de transporte motorizado, devido à distância, para chegar à unidade de saúde da APS?
	Os pais/cuidadores da pessoa com hanseníase perdem turno de trabalho ou compromissos e a criança/adolescente falta à aula para ser atendido na unidade de saúde?	Os pais/cuidadores faltam ao trabalho ou compromissos para que a criança/adolescente com hanseníase seja atendida na unidade de saúde?
	A unidade de saúde dispõe de meios para se comunicar com o usuário/paciente (exemplo: telefone, mensagens de texto SMS, mensagens por aplicativo como *WhatsApp*, e-mail etc.) sobre o tratamento, confirmação de consultas etc.?	A unidade de saúde dispõe de meios para se comunicar com o usuário/paciente (exemplo: telefone, mensagens de texto SMS, mensagens por aplicativos como *WhatsApp*, e-mail etc.) sobre o tratamento, confirmação de consultas etc.?
Atendimento continuado	Você conhece toda a história de saúde da criança/adolescente com hanseníase?	Você está familiarizado com os antecedentes de saúde da criança/adolescente com hanseníase?
	Você questiona aos pais/cuidadores da criança/adolescente como a hanseníase impacta a vida cotidiana, se há preconceito por parte de outras pessoas ou se a doença afeta as atividades escolares e de lazer?	Você questiona os pais/cuidadores/adolescente sobre como a hanseníase afeta a vida diária e interfere nas atividades escolares e de lazer?
Orientação familiar	Você discute com os pais/cuidadores da criança/adolescente sobre as técnicas de autocuidado para a prevenção de incapacidades?	Você fornece orientações aos pais/cuidadores da criança/adolescente sobre os sinais e sintomas das reações hansênicas, além de instruir sobre técnicas de autocuidado para a prevenção de incapacidades?

## DISCUSSÃO

O processo de validação de conteúdo é crucial na elaboração de medidas e instrumentos na área da saúde, permitindo uma avaliação e prática mais seguras, livres de erros de mensuração. Esse processo engloba conceitos teóricos e aspectos práticos vinculados ao julgamento e à percepção de um grupo de especialistas com conhecimento prévio no assunto a ser validado, com o objetivo final de se obter um instrumento capaz de mensurar precisamente o que se propõe^(19)^.

A validação do Instrumento para Avaliação da Qualidade da Atenção à Criança e Adolescente com Hanseníase no Contexto da APS, proposta nesta pesquisa, estabelece um marco importante para a avaliação dos atributos essenciais e derivados na APS. O instrumento foi avaliado por juízes com expertise no tema, majoritariamente enfermeiros, com doutorado e experiência em ensino e pesquisa.

Quanto aos itens avaliados, segundo os atributos essenciais e derivados, o Índice de Validação de Conteúdo (IVC) total foi de 0,98, e os atributos Porta de Entrada e Orientação Comunitária obtiveram os maiores escores, o que confere valores satisfatórios que asseguram parâmetros de validade em relação ao conteúdo utilizado para a elaboração dos itens de cada atributo do instrumento.

As análises de validação de conteúdo refletem um processo essencial no desenvolvimento de instrumentos de avaliação de serviços, especialmente em populações mais vulneráveis. O aumento dos casos de hanseníase nessa faixa etária indica a persistência endêmica da condição na comunidade. Este procedimento é vital para evitar a ocorrência de resultados e medidas imprecisos ou tendenciosos que possam levar a conclusões errôneas ou mal mensuradas.

Em relação à amostra de juízes para a avaliação do instrumento em serviços de saúde, defende-se que essa amostra seja multiprofissional, pois profissionais de diferentes áreas podem contribuir com perspectivas, ideias e experiências diversas, aumentando o rigor, critério e objetividade na avaliação do fenômeno e do conteúdo validado^(20)^. Neste estudo, os juízes foram principalmente enfermeiros e médicos, que são, na maioria das vezes, os profissionais que prestam assistência à hanseníase na APS e, portanto, possuem maior capacidade para avaliar os atributos essenciais e derivados no atendimento à população estudada.

Quanto ao número de juízes para a técnica Delphi na validação de conteúdo, diversas instituições e autores concordam que o número mínimo adequado deve ser de pelo menos sete especialistas, uma vez que isso reduz a probabilidade de erros na aferição. Menciona-se também que o número máximo de juízes não deve ultrapassar trinta participantes, pois os benefícios observados com um número maior não são significativos. Assim, o número de juízes utilizado neste estudo foi considerado satisfatório, estando dentro do mínimo e máximo recomendados pela literatura^(21-22)^.

Quanto ao fato de a amostra de juízes ter sido composta por profissionais especialistas, mestres e doutores atuantes como docentes, pesquisadores ou trabalhadores da assistência em saúde, é relevante destacar que a escolha se baseou em profissionais com experiência na temática de interesse, reduzindo consideravelmente possíveis inconsistências nos resultados^(23-25)^.

A validação da versão para profissionais de saúde do instrumento de avaliação do desempenho da APS nas ações de controle da hanseníase em população adulta (PCAT - hanseníase) corrobora com o presente estudo, indicando valores satisfatórios em termos de parâmetros de validade, consistência interna e confiabilidade, assegurando sua utilidade nos processos avaliativos dos serviços de saúde^(12)^.

É importante a validação de instrumentos que utilizam os atributos da APS para a avaliação de serviços, tanto em uma perspectiva geral quanto em doenças específicas. Destaca-se a necessidade de medir o grau de orientação dos serviços dentro de um sistema que prioriza a APS no modelo assistencial, focando em parâmetros avaliativos que identifiquem os aspectos que exigem maiores investimentos ou a reorientação de ações no sistema de saúde^(26)^.

O atributo Porta de Entrada, neste estudo, apresentou escore satisfatório na validação de conteúdo, sendo considerado essencial como componente do acesso de primeiro contato, crucial para a interface entre usuário e serviço de saúde, abrangendo as demandas espontâneas e o agendamento de atendimentos^(27-29)^.

Investir em ferramentas e ações que facilitem o acesso dos usuários aos serviços de saúde, especialmente na população infantil, é vital. Nessa perspectiva, o acesso e a acessibilidade aos serviços, como a prevenção da hanseníase, busca ativa de casos, imunoquimioprofilaxia, diagnóstico precoce, rastreamento por técnicas inovadoras de biologia molecular e tratamento oportuno, são fundamentais^(30-32)^.

Além disso, questões relacionadas ao tempo de espera por atendimento, distância geográfica do estabelecimento de saúde, horários e turnos de funcionamento, que poderiam estar alinhados ao funcionamento de creches e instituições de ensino, são essenciais para potencializar uma assistência mais qualificada. Nessa perspectiva, o Acesso é visto como principal atributo essencial da APS, sendo a ponte entre o sistema e o usuário e garantindo equidade e qualidade na prestação dos serviços^(33)^.

Em relação à Longitudinalidade do Cuidado ou Atendimento Continuado, destaca-se na literatura o grande potencial desse atributo dentro do contexto da APS, uma vez que possibilita o conhecimento profundo do usuário em seu contexto social, sua história de vida, hábitos e costumes. Isso permite planejar e estabelecer planos de cuidados individuais ou coletivos, proporcionando intervenções adequadas e resolutivas. Observa-se que a Estratégia de Saúde da Família (ESF) apresenta melhores valores na avaliação do Atendimento Continuado em comparação às unidades básicas de saúde tradicionais, sendo considerado um modelo assistencial promotor do cuidado longitudinal, o que pode impactar positivamente na qualidade da atenção à saúde prestada^(34-35)^.

A Integralidade dos Serviços Disponíveis e Prestados, como atributo da APS, visa valorizar a qualidade da sistematização da assistência, especialmente no protocolo de atendimento à hanseníase em crianças e adolescentes, considerando também os aspectos biopsicossociais associados a essa clientela^(5,36-39)^.

Por sua vez, o atributo Coordenação do Cuidado tem como objetivo estabelecer conexões em uma rede de serviços dentro do sistema de saúde, alcançando o Atendimento Integral e promovendo a Longitudinalidade do Cuidado em Saúde. Este atributo fortalece aspectos que favorecem a qualidade do serviço e da atenção prestada, visando reduzir as barreiras de acesso a diferentes níveis de atenção e integrar o máximo possível de serviços dentro de um mesmo território, mesmo que em contextos diversos. A Coordenação do Cuidado instrumentaliza mecanismos que correlacionam a organização das redes de atenção à saúde, em um processo de regionalização, buscando aprimorar os processos por meio da redução de filas, priorização de necessidades, e elaboração de protocolos e fluxogramas que visam padronizar a qualidade do serviço prestado^(40-41)^.

Além disso, estudos que objetivaram a validação de instrumentos para a avaliação da coordenação do cuidado e a ordenação nas redes de atenção pela APS demonstram que a maioria dos itens propostos alcançou um grau de estabilidade suficiente, com alto consenso entre os especialistas envolvidos no processo de validação de conteúdo, ressaltando a importância dos itens que avaliam a continuidade das ações de saúde prestadas por diferentes profissionais em diferentes pontos da rede de atenção^(42-43)^.

Por fim, a Orientação Familiar, como atributo da APS, deve basear-se em um modelo assistencial centrado na família, permitindo uma análise ampliada das questões relacionadas ao processo saúde-doença, através de uma hermenêutica socioespacial, focada na reorganização dos serviços e direcionada especialmente para a ESF enquanto coordenadora do cuidado familiar, respeitando os preceitos de universalidade, igualdade e equidade do sistema de saúde. No contexto da saúde da criança e do adolescente, esse atributo é ainda mais relevante, pois o cuidado a essa clientela é influenciado pela percepção familiar das ações de promoção, prevenção e do processo de adoecimento^(44-45)^.

A aproximação da comunidade aos serviços e decisões em saúde é um grande desafio a ser superado no contexto da APS. É imperativo encontrar mecanismos para que as unidades de APS estejam orientadas à comunidade, estabelecendo uma conexão entre o serviço, os profissionais de saúde e os usuários presentes no espaço geográfico ou no contexto social e político que define o território^(36,46)^.

Neste contexto, a orientação profissional tem como objetivo entender a percepção dos profissionais sobre suas próprias qualificações no atendimento aos problemas de saúde, sendo um fenômeno central para avaliação. Este atributo abrange questões cruciais relacionadas à formação profissional e à educação continuada. Assim, ressalta-se a importância do investimento na formação e capacitação de médicos, enfermeiros e outros profissionais de saúde que atendem crianças e adolescentes. É vital estabelecer uma parceria com instituições de ensino, promover a valorização profissional, aprimorar a estrutura física dos serviços e investir em ferramentas tecnológicas inovadoras para a implementação de práticas assistenciais^(12,47)^.

Na validação do PCATool Brasil versão adultos, observa-se que o processo de validação de conteúdo não adotou a técnica Delphi, impossibilitando comparações entre os dois modelos^(48)^.

O instrumento em questão foi empregado em um estudo em um município do estado do Pará, revelando-se uma ferramenta adequada para cumprir seu propósito de medição^(49)^.

### Limitações do estudo

Este estudo apresenta limitações. Primeiramente, a amostra de juízes, apesar de ser qualificada e diversificada, é limitada em número e pode não representar todas as perspectivas e experiências dos profissionais de saúde que atuam na APS em diferentes regiões e contextos. Além disso, o processo de validação de conteúdo, embora rigoroso, pode ter sido influenciado pela subjetividade dos juízes na interpretação dos itens do instrumento.

### Contribuições para a Área da Enfermagem e Saúde Pública

Embora existam limitações, a validade de conteúdo é fundamental no processo de desenvolvimento e adaptação de instrumentos de medida. A inclusão de questões validadas sobre agravos ou políticas de saúde nos instrumentos oficiais de avaliação do Ministério da Saúde representa um grande avanço na busca por uma adequada mensuração na política avaliativa da saúde no país. Considerando que a avaliação em saúde é uma medida necessária no processo de melhoria da qualidade prestada, o instrumento validado quanto ao conteúdo proposto neste estudo pode contribuir de maneira satisfatória na avaliação das ações na assistência à hanseníase no contexto da APS, especialmente no atendimento a crianças e adolescentes com hanseníase.

## CONCLUSÕES

Este estudo teve como propósito principal validar o conteúdo do instrumento para avaliação da qualidade da atenção à criança e ao adolescente com hanseníase no contexto da Atenção Primária à Saúde. Os resultados obtidos evidenciam que o instrumento proposto alcançou um alto nível de validade de conteúdo, conforme indicado pelo Índice de Validação de Conteúdo e valores satisfatórios de confiabilidade, medidos pelo Alfa de Cronbach.
